# The correlation between human papillomavirus and oral lichen planus: A systematic review of the literature

**DOI:** 10.1002/iid3.960

**Published:** 2023-08-08

**Authors:** Farzaneh Agha‐Hosseini, Kimia Hafezi Motlagh

**Affiliations:** ^1^ Dental Research Center Dentistry Research Institute, Tehran University of Medical Sciences Tehran Iran; ^2^ Department of Oral Medicine, Faculty of Dentistry Tehran University of Medical Sciences Tehran Iran; ^3^ The Academy of Medical Sciences Tehran Iran; ^4^ Department of Oral Medicine, School of Dentistry Tehran University of Medical Sciences Tehran Iran

**Keywords:** human papillomavirus, lichen planus, oral lichen planus

## Abstract

**Introduction:**

Oral lichen planus (OLP) is a chronic inflammatory disorder with cell‐induced immunopathological responses and is considered a potential malignancy disorder in the oral cavity. Due to the high prevalence of OLP as well as the potential for malignancy, human papillomaviruses (HPVs) may play an important role in it. Although previous studies have explored the possible relationship between HPV and OLP, the findings have been conflicting and nonconclusive. This study aims to review the studies that investigated HPV‐16 and HPV‐18 in OLP.

**Methods and Materials:**

The research protocol followed the Preferred Reporting Items for Systematic Reviews (PRISMA2020) checklist. The online databases Pubmed, Scopus, Embase, Google Scholar, and Cochrane were searched using the following individual keywords: “OLP” OR “Oral Lichen Planus” OR “HPV” OR “Human Papillomavirus.” The search strategy resulted in the selection of 80 articles. The articles were evaluated, and after duplication removal, 53 abstracts were reviewed, resulting in the selection of 25 studies according to inclusion and exclusion criteria. The risk of bias assessment was done by using the Modified Newcastle–Ottawa quality assessment scale. The overall prevalence of HPV in OLP lesions varied from 2.7% to 70%, depending on the type of diagnostic method used.

**Conclusion:**

Despite the studies conducted on the relationship between OLP and HPV infection, there is still no conclusive evidence that HPV can play a role in the etiopathogenesis of OLP, either in clinical manifestations or in the malignant transformation of lesions.

## 1 INTRODUCTION

Oral lichen planus (OLP) is a chronic inflammatory disorder with cell‐induced immunopathological responses and is considered a potential malignancy disorder (PMD) in the oral cavity. The common term (OPMD) of potentially malignant oral disorders is suggested for oral premalignancy, including premalignant lesions (oral leukoplakia, erythroplakia, and proliferative verrucous leukoplakia) and premalignant conditions (lichen planus and submucosal fibrosis). All oral mucosa lesions with the risk of malignant changes are included under the term OPMD.^1^ Its exact etiology is unknown, but there is strong evidence confirming the role of lymphocytes in the pathogenesis of this disorder.^2^ Immune system deficiency plays a primary role in the development of this disease, and it is currently considered a disorder mediated by autoimmune cells. Clinically, OLP has six different types: papule, reticular, plaque, atrophic, erosive, and bullous, and the most common type is reticular. All types of OLP can be classified into two general clinical groups: erosive‐atrophic forms (EA‐OLP), including erosive, atrophic, and bullous types; and non‐erosive‐atrophic types (non‐EA‐OLP), including papular, reticular, plaque‐like types. EA‐OLP are more prone to malignant changes than non‐EA‐OLP.^3^ It affects 0.5%–2.2% of the population, which varies depending on the geographic location, and the ratio of females to men is reported to be 1.4–1.^4^

OLP mostly occurs in middle‐aged adults and may occur in any area in the oral cavity, but the buccal mucosa, tongue, and gingiva are the most common areas of involvement.^5^ The clinical appearance of OLP characteristics is multiple keratotic papules and reticular lines with bilateral and symmetrical distribution, which can also appear as desquamative gingivitis.^6^ OLP lesions appear as inflamed sores that may have a white linear pattern. Usually, the presence of lesions is persistent, although lesions do not remain in one area of the mouth or skin and tend to migrate over time. Lesions are recognized by their periods of recovery and flare‐ups.^7^ When OLP is present, it is challenging for the patient to eat, drink and function due to persistent pain. This condition can be triggered by physical or psychological stress, the presence of chronic mechanical trauma, and the consumption of certain stimulant foods and drinks.^8^ The diagnosis of OLP and other disorders with similar clinical appearance, such as oral lichenoid lesions, is very important. Although OLP has been studied for many decades, its exact etiology is still unknown. In recent articles autoimmunity, stress, trauma, viruses such as hepatitis C (HCV),^9^ herpes simplex virus (HSV),^10^ Epstein‐Barr virus^11^ have been proposed as effective factors in the etiology of OLP. OLP, in general, is known as an immunological process in which the involvement of specific and nonspecific antigens is involved in its pathogenesis and histologically, it shows the appearance of a hypersensitivity reaction.^12^

The factors that initiate OLP are unknown, but its mechanism includes several steps, including an initiating factor or a phenomenon (exogenous/endogenous antigenic stimulation), local release of regulatory cytokines, upregulation in vascular adhesion molecules, accumulation, and retention of T lymphocytes and cytotoxicity in basal keratinocytes regulated by T lymphocytes.^13^

In 1997, the World Health Organization classified OLP as a potentially malignant disorder with a nonspecific risk of malignant transformation and suggested that patients should be closely monitored. In a meta‐analysis conducted by Syrjänen, a strong relationship between HPV and oral squamous cell carcinoma and other premalignant conditions has been suggested. HPV16 levels were higher in OLP patients compared to healthy controls. These findings suggest that HPV may play a role in the etiology and malignant changes of lichen planus.^14^ Recently, it has been found that viruses such as human papillomavirus (HPV) and human herpes simplex virus (HSV) are involved in the pathogenesis of OLP. Available data suggests that these viruses may alter host cell function by inducing abnormal expression of cellular proteins and leading to disease progression. It is not easy to explain why a chronically inflamed epithelium can be attacked by T cells and become susceptible to HPV infection. An explanation for this phenomenon can be that the wounds that are often present in OLP, especially in its atrophic types, make the mucosa more susceptible to HPV infection. Another explanation could be the chronic use of steroids, where the immunosuppression induced by the use of these drugs may lead to upregulated HPV transcription.^15^

HPV is a member of the Papillomavirus family, with a diameter of 50–500 nm and no capsular coating. Different types of HPV are recognized based on the degree of nucleic acid sequence homology. Different types of HPV, including HPV‐16, HPV‐18, and HPV‐31, have been associated with certain types of malignant and premalignant lesions.^16^ HPV is a small circular DNA virus that contains three genomic regions: the initial region (E), the late region (L), and the long control region (LCR). Based on oncogenic characteristics, HPVs are divided into two groups: high‐risk HPVs and low‐risk HPVs. High‐risk HPVs have a high ability to cause malignant changes in mucosal epithelial cells. The most common high‐risk HPV in the oral mucosa is HPV‐16.^17^ HPV‐16 and HPV‐18 have been reported significantly with PMD (premalignant disorders) and OSCC (oral squamous cell carcinoma).^18^

The common role of HPV has been reported in OLP and OSCC, but there are wide differences in its prevalence depending on different geographic populations. In addition, data from the International Biological Study of Cervical Cancer has shown that phylogenetic clusters of HPV16 are in direct association with specific racial groups and geographic locations. Most HPV infections are asymptomatic, although the lesions can occur at any time after infection. The period of HPV infection depends on its subtype and related factors such as smoking, nutritional status, immune status, and hormonal effects.^13^ HPV virus, specifically targets undifferentiated basal cells and proliferative mucosa.^19^ Aberrant and continuous gene expression of E6 and E7 proteins in high‐risk HPVs leads to genomic instability and the occurrence of mutations, which can cause malignant changes due to infection.^20^ Low‐risk genotypes, such as HPV‐6/‐11, can cause benign anogenital and cutaneous warts, while high‐risk genotypes, including HPV‐16/‐18, play an important role in tumor progression.

Due to the high prevalence of OLP and the painful and irritating nature of erosion atrophic types of this disease, as well as the potential of malignancy and the formation of oral squamous cell carcinoma in some types of OLP, HPV‐16 and HPV‐18 may play an important role in it. This review study aims to review the studies that investigated HPV‐16/‐18 in OLP.

## 2 METHODS

### 2.1 Search strategy

Searching in Electronic databases, Ovid, Embase, Pubmed, Google Scholar, Scopus, and Cochrane were searched from 1960 to March 2022 with the keywords “OLP” OR “Oral Lichen Planus” AND “HPV” OR “Human Papillomavirus”. In addition, all the references of these articles were checked manually to identify other relevant articles that were not identified in the initial search. Ethics approval and patient informed consent were not required as this study is a review based on published literature.

### 2.2 Eligibility criteria

The studies selected in this study included case–control studies and clinical trials with the topic of examining the HPV‐16/‐18 virus in tissue samples of oral mucosa and saliva of people with OLP. Only studies published in English were included in this study. Studies were excluded from our study due to the unavailability of the full text, irrelevant results, lack of WHO criteria to approve the OLP, studies without a defined control group, and systematic review articles.

### 2.3 Data extraction

At this stage, both researchers (FA and KHM) individually examined the obtained articles in three steps. In the first step, using Endnote 20 software and then manual review, duplicate sources were removed. In the second step, articles were screened by examining their titles and abstracts to exclude studies not related to the present study. In the third full‐text step, the articles were studied in full, and their eligibility was checked based on the determined entry and exclusion criteria.

A data extraction sheet was developed by using Microsoft Excel 2013 software. Two authors have independently extracted study characteristics from the included article including: author name, publication year, aim of study, study design, sample size, detection methods of HPV, and subtype of OLP if applicable.

## 3 RESULTS

A total of 80 articles were obtained from five electronic databases: Pubmed, Scopus, Embase, Google Scholar, and Cochrane, and by an additional manual search of the sources of the selected articles, an article was added in total. After removing duplicate articles, 55 articles entered the next stage of Screening (*n* = 55). At this stage, by screening studies based on title and abstract, two studies were excluded due to not being in English (*n* = 53).

From the remaining 53 studies that were examined to verify eligibility for full‐text reading, 16 studies were excluded (5 studies due to unavailability, 1 study due to the irrelevant results with the study title, and 10 studies due to systematic review were deleted). From the remaining 37 studies, 12 studies were excluded due to the lack of a control group, the lack of WHO criteria to confirm OLP, and the indirect effect of HPV on OLP (Table 1), and as a result, the final number of 25 studies were included to participate in this review (*n* = 25). Of a total of 25 studies, 23 were case–control studies and 2 were cohort studies, all of which were performed with the aim to investigate the prevalence of HPV in OLP.

**Table 1:** The excluded studies and the reason of exclusion.

Year/author	Reason of exclusion
Vesper et al. 1997^43^	Full text was not in English.
Bouda et al. 2000^44^	Aimed to investigate the role of HPV in oral potentially malignant lesions but not include OLP.
Dodson 2010^45^	Full text was not available.
Montebugnoli et al. 2014^46^	Not assessing the direct relationship between HPV and OLP.
Viguier et al. 2015^47^	Not assessing the direct relationship between HPV and OLP.
Nafarzadeh et al. 2017^48^	Full text was not available.

**Figure 1:**
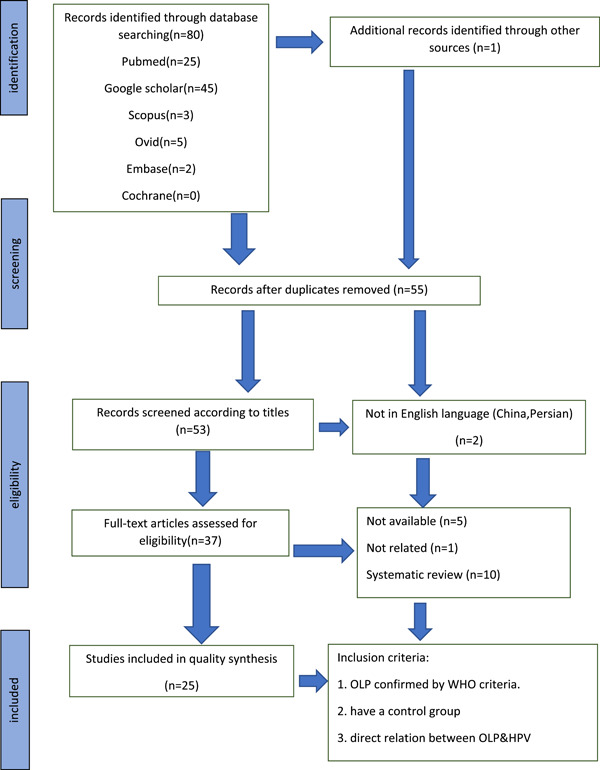
PRISMA 2020 flow diagram.

The reasons for excluding studies are summarized in the PRISMA chart (Figure 1). The details of each of the final selected studies are listed in Table 2.

**Table 2:** Details characteristics of the included studies.

Author/year	Aim of study	Type of HPV	Detection method	Study design	Samples	Number of cases/male/female	HPV prevalence
Syrjänen 1986^21^	TO detect human papillomavirus (HPV) DNA of types 6, 11, and 16 in paraffin sections of oral lesions (leukoplakia, oral lichen planus [OLP], SCC)	16	In situ DNA hybridization	Case–control	Tissue samples (FFPE)	32 cases/18 males/14 females	1 of 32 cases (3.2%)
Jontell et al. 1990^22^	To identify HPV in erosive OLP	6, 11, 16, 18	PCR	Case–control	Biopsy from patients	20 cases/3 males/17 females	13 of 20 samples (65%) were positive for some HPV type. 25% HPV‐6 15% HPV‐16
Sand et al. 2000^23^	To identify an association between HPV infection and oral lesions such as OLP.	6, 11, 16, 18	PCR	Case–control	Biopsy from patients	65 cases (53 oral lesions and 12 control)	6 of 22 (27.3%) were HPV‐positive, 5 for HPV‐18, and 1 for nonspecific primer
ÓFlatharta et al. 2003^10^	To ascertain if the human herpesviruses or human papillomaviruses act as possible factors in the pathogenesis of OLP	16	PCR and Southern blot	Case–control	Tissue samples (FFPE)	58 cases (38 cases of OLP and 20 control)	HPV‐16 was detected in 26.3% of OLP samples
Ostwald et al. 2003^24^	An association of oral leukoplakia, oral SCC, and OLP and infection with the high‐risk HPV types 16 and 18.	6, 11, 16, 18	PCR/Southern blot hybridization	Case–control	Tissue samples (FFPE)	118 oral squamous cell carcinomas, 72 oral leukoplakias, and 65 OLP	HPV‐16 and HPV‐18 DNA were present in 6 of 65 (9.2%) lichen planus
Campisi et al. 2004^25^	To determine the prevalence of HPV infection in oral leukoplakia (OL) and OLP in comparison with that in healthy oral mucosa	16, 18	PCR	Case–control	Biopsy from patients	139 cases/66 males/73 females	18.5% of non‐atrophic‐erosive/20.4% of atrophic‐erosive OLP
Giovannelli et al. 2006^26^	Detection of HPV in oral leukoplakia, OLP, and oral SCC by brushing of Oral Mucosa	18	PCR	Case–control	Brushing the mucosal surfaces	50 patients with OL, 49 with OLP, and 17 with OSCC	24.5% of OLP
Razavi et al. 2009^27^	To examine the coincidence of human papillomavirus type 18 and OLP	18	PCR	Case–control	Tissue samples (FFPE)	29 cases	31.0% of OLP
Szarka et al. 2009^28^	We aimed to examine the effect of HPV carriage on the risk of the development of potentially malignant lesions	16, 18	PCR	Case–control	Biopsy from patients	31 males/88 females	EA‐OLP (42.6%) Non‐EA‐OLP (22.4%) HPV‐16 (23 of 119) HPV‐18 (5 of 119)
Ali 2009^29^	To describe an association of high risk‐HPV genotypes with a variety of oral lesions including squamous cell carcinoma, leukoplakia, and lichen planus	16, 18	In situ hybridization	Case–control	Tissue samples (FFPE)	42 cases	33.3% HPV positive in OLP. HPV‐16‐DNA in all HPV‐positive OLP tissues. None of these OLP tissues showed HPV‐18 positivity.
Yildirim et al. 2011^30^	To assess the prevalence of Herpes Simplex virus, Epstein Barr virus, and Human Papillomavirus‐16 in OLP	16	Immunohistochemically	Case–control	Biopsy from patients	65 cases	14 of 65 cases (21%)
Mattila et al. 2012^15^	We assessed HPV‐genotype distribution in atrophic OLPs	16	Luminex‐based assay	Case–control	Biopsy from patients	82 cases	HPV DNA was found in 15.9% of OLPs
Kato et al. 2013^31^	We assessed HPV‐genotype distribution in OLPs of Japanese patients	16, 18	PCR and direct DNA analysis.	Case–control	Biopsy from patients	200 cases/77 males/123 females	HPV‐16 (36.0%) and HPV‐18 (32.0%)
Arirachakaran et al. 2013^16^	To explore the association between HPV and OLP in Thai patients	16	PCR	Case–control	Tissue samples (FFPE)	37 cases	HPV DNA was detected in one tissue biopsy from an atrophic‐type OLP (1 of 37)
Sahebjamiee et al. 2015^32^	To assess the prevalence of high‐risk HPV‐16 and HPV‐18 in tissue and saliva samples from an Iranian population diagnosed with OLP	16, 18	PCR	Cohort	Biopsy from patients	40 cases/8 males/32 females	HPV‐16 and HPV‐18 were positive in 8 of 40 (20%) OLP tissues
Pol et al. 2015^33^	To assess the prevalence of HPV‐16 in histopathologically diagnosed specimens of OLP	16	Immunohistochemical	Case–control	Biopsy from patients	60 cases/25 males/35 females	21 in 30 cases (70%)
Cao et al. 2016^34^	Determination of human papillomavirus in oral leukoplakia, OLP, and oral squamous cell carcinoma	16, 18	In situ hybridization	Case–control	Tissue samples (FFPE)	255 cases	8 of 66 cases (12.12%)
Liu et al. 2018^35^	To investigate the expressions of p16 and HPV16/18 (E6) in OLP and malignant transformed OLP (MT‐OLP)	16, 18	Immunohistochemical	Case–control	Biopsy from patients	46 cases (40 cases of OLP and 6 MT‐OLP)	HPV infection rates in OLP and MT‐OLP were 67.50% and 100.00%
Gomez‐Armayones et al. 2019^36^	To evaluate the HPV‐DNA prevalence and type distribution in a set of oral biopsies obtained from patients diagnosed with OLP and dysplasia	16, 18	PCR	Retrospective cohort	Tissue samples (FFPE)	100 cases	Two samples were HPV‐16 positive (2%) and one sample was HPV‐18 positive (1%)
Sameera et al. 2019^3^	To investigate the possibility of HPV presence in OLP	18	PCR	Case–control	Biopsy from patients	30 cases	86.6% of HPV‐18 positive
Farhadi et al. 2020^37^	To evaluate the prevalence of HPV‐16, HPV‐18, and HPV‐33 in erosive lichen planus	16, 18	PCR	Case–control	Biopsy from patients	32 OLP samples (consisting of 12 reticular and 20 erosive forms of OLP)	8 of 32 cases (25% were HPV positive and 3.12% were HPV‐18 positive)
Kaewmaneenuan et al. 2021^38^	The detection rate of high‐risk human papillomavirus types 16 and 18 in oral potentially malignant disorders (OPMDs) in a Thai population	16, 18	PCR	Case–control	Tissue samples (FFPE)	160 (101 OL and 59 OLP)	18.6% HPV‐positive in OLP. HPV‐18 DNA was predominantly detected in both OL and OLP patients (90%).
Bar et al. 2021^39^	To assess p53 protein, HSP90, and E‐cadherin expression and presence of HPV16/18 in two subtypes of OLP	16, 18	Immunohistochemical	Case–control	Biopsy	122 cases/65 males/57 females	4 of 56 (7.1%) cases were HPV‐16/18/positive
Della Vella et al. 2021^40^	To evaluate the prevalence of HPV infection in a cohort of patients affected by OLP	16	PCR	Case–control	Biopsy from patients	52 cases	The total prevalence of HPV infection was 17%. Hyperkeratotic OLP was 19%, while in the erosive forms was 11%.
Saber et al. 2022^41^	To determine the prevalence of co‐infection with HPV, Epstein –Barr virus, and Merkel Cell PolyomaVirus in OLP and SCC	16, 18	PCR	Cross‐sectional	Tissue samples (FFPE)	114 extracted DNA	46.2% of erosive OLP, 18.8% of reticular OLP were HPV‐positive

Results of this study showed that among the articles reviewed in this review, 14 (56%) articles with PCR method, 4 (16%) articles with immunohistochemistry (IHC) method, 3 (12%) articles with in situ hybridization (ISH) method, 3 (12%) articles with both PCR and Southern blot methods, and 1 (4%) article with Luminex‐based method had identified the HPV virus. In terms of the type of samples, the results showed that 56% of the studies were conducted on fresh biopsy of the oral mucosa and 40% of the studies on formalin‐fixed paraffin‐embedded (FFPE) blocks, and 4% were conducted with the brushing method of oral mucosa cells.

### 3.1 Risk of bias assessment

Risk of bias assessment was done by the two authors using Modified Newcastle–Ottawa quality assessment scale^42^ (Table 3 and 4).

**Table 3:** Modified Newcastle–Ottawa quality assessment scale for case–control studies.

Study	Factor								
	Selection				Comparability	Exposure			Modified Newcastle Ottawa score (Risk of Bias)
	Is the case definition adequate?	Representativeness of the cases	Selection of controls	Definition of controls	Comparability of cases and controls on the basis of the design or analysis	Ascertainment of exposure	Same method of ascertainment for cases and controls	Nonresponse rate	
Syrjänen 1986^21^	No	No	Yes	No	Yes	Yes	Yes	Yes	5
Jontell et al. 1990^22^	Yes	Yes	Yes	No	Yes	N/A	Yes	No	5
Sand et al. 2000^23^	Yes	Yes	Yes	Yes	Yes	Yes	Yes	No	7
ÓFlatharta et al. 2003^10^	Yes	Yes	Yes	Yes	Yes	Yes	Yes	No	7
Ostwald et al. 2003^24^	Yes	Yes	Yes	Yes	Yes	Yes	Yes	No	7
Campisi et al. 2004^25^	Yes	Yes	Yes	Yes	Yes	Yes	Yes	No	7
Giovannelli et al. 2006^26^	Yes	Yes	Yes	Yes	Yes	Yes	Yes	No	7
Razavi et al. 2009^27^	Yes	Yes	Yes	Yes	Yes	Yes	Yes	No	7
Szarka et al. 2009^28^	Yes	Yes	Yes	Yes	Yes	Yes	Yes	No	7
Ali 2009^29^	Yes	Yes	Yes	Yes	Yes	Yes	Yes	No	7
Yildirim et al. 2011^30^	Yes	Yes	Yes	No	Yes	Yes	Yes	N/A	6
Mattila et al. 2012^15^	Yes	Yes	Yes	Yes	Yes	Yes	Yes	Yes	8
Kato et al. 2013^31^	Yes	Yes	Yes	Yes	Yes	Yes	Yes	N/A	7
Arirachakaran et al. 2013^16^	Yes	Yes	Yes	No	Yes	Yes	Yes	No	6
Pol et al. 2015^33^	Yes	Yes	Yes	Yes	Yes	Yes	Yes	No	7
Cao et al. 2016^34^	Yes	Yes	Yes	No	Yes	Yes	Yes	No	6
Liu et al. 2017^35^	Yes	Yes	Yes	Yes	Yes	Yes	Yes	Yes	8
Sameera et al. 2019^3^	Yes	Yes	Yes	No	Yes	Yes	Yes	No	6
Kaewmaneenuan et al. 2021^38^	Yes	Yes	No	No	Yes	Yes	Yes	N/A	5
Farhadi et al. 2020^37^	Yes	Yes	Yes	Yes	Yes	Yes	Yes	N/A	7
Bar et al. 2021^39^	Yes	Yes	Yes	Yes	Yes	Yes	Yes	No	7
Della Vella et al. 2021^40^	Yes	Yes	Yes	Yes	Yes	N/A	Yea	No	6

**Table 4:** Modified Newcastle–Ottawa quality assessment scale for cohort studies.

Study	Selection				Comparability	Outcome			Score
	Representativeness of the exposed cohort	Selection of the nonexposed cohort	Ascertainment of exposure	Demonstration that outcome of interest was not present at the start of the study	Comparability of the cohort based on the design or analysis	Assessment of outcome	Was follow‐up enough for outcomes to occur?	Adequacy of the follow‐ups of cohorts	
Sahebjamiee et al. 2015^32^	Yes	Yes	Yes	N/A	Yes	Yes	N/A	N/A	5
Gomez‐Armayones et al. 2019^36^	Yes	N/A	Yes	N/A	Yes	Yes	Yes	N/A	5

## 4 DISCUSSION

OLP is a chronic inflammatory disorder with periods of relapse and remission and with the potential of malignant transformation (MT).^49^, ^50^ The prevalence of this disorder in the general population is approximately 1%–2%. One of the important and debatable points about OLP is its malignant potential, especially in its erosive form. In general, OLP has a malignant transformation rate between 0.5% and 1.2%, which has been reported up to 2.8% in some studies.^51^ A recent meta‐analysis showed that 6% of dysplastic OLP samples progress to OSCC, although less than 1.5% of OLP samples without dysplasia eventually progress to OSCC.^52^ The exact etiology of OLP is still unknown, but it is known in scientific sources as an inflammatory and autoimmune lesion that is initiated by an antigen and exposes keratinocyte cells of the basal layer to immune responses.^37^ This process causes the activation of CD4 and CD8 lymphocytes as well as the production of cytokines such as interleukin‐2 (IL‐2), interferon‐gamma, and TNF, which induce apoptosis of keratinocyte cells. The antigen causing the initiation of these immune responses can be internal or external.^53^

For the first time in the 1987s, the relationship between HPV and OLP was proposed.^54^ Many researchers have studied the role of HPV in OLP lesions and reported various findings related to the prevalence of this virus and its subtypes in OLP.

HPV is the second most common sexually transmitted infection, of which more than 200 types are known to date. This virus can cause cancer of the vagina, penis, anus, and some cancers of the head and neck, especially the oropharynx. Infection with HPV can cause various skin manifestations, including (1) common warts, (2) stringy warts, and (3) warts on palms and soles. HPV16 accounts for approximately 95% of HPV‐positive oropharyngeal carcinomas.^55^ HPV is a small, non‐enveloped, circular double‐stranded deoxyribonucleic acid (DNA) virus, 52–55 nm in diameter. This double‐stranded DNA contains approximately 8000 base pairs, which are attached to cellular histones. The HPV genome encodes eight open reading frames (ORFs). The primary ORFs include E1, E2, E4, E5, E6, and E7 proteins that control viral replication. The terminal ORF contains structural proteins L1 and L2. L1 protein and L2 capsid protein help to deliver the viral genome into the epithelial cells. Based on the E6 and E7 nucleotide sequence, HPV viruses are classified into two low‐risk and high‐risk categories.^56^, ^57^

Among the studies we examined in this review, the overall prevalence of HPV in OLP lesions has been reported from 2.7%^16^ to 70%^33^ according to the type of diagnostic method used. HPV16 is the most common genotype reported, and HPV18 is the second most common genotype in OLP patients.^58^ Among all these articles, four studies examined the incidence of HPV in relation to the clinical type of OLP, and HPV‐positive samples were reported in erosive atrophic lichen planus (EA‐OLP). In one of the studies examined in this review, the relationship between severe erosive lichen planus and HPV infection was raised, and the authors proposed the hypothesis that the HPV16 vaccine, which has a protective role in cervical cancer, may protect against erosive lichen planus.^47^ It seems that more studies are needed to investigate the prevalence of HPV infection in each of the clinical subtypes of OLP.

To better understand the process of HPV carcinogenesis, we must know that HPV shows a preference for squamous epithelium, and the abraded and scraped epithelium transmits the virus. To develop a fixed infection with HPV, the virions must reach the basal lamina in the squamous epithelium through the microlesions in the epithelium. The main capsid protein L1 and the partial capsid protein L2 deliver the virus genome to the nucleus of epithelial cells.^59^ E1 and E2 are the first viral proteins to be expressed. E5 protein, which plays an important role in the productive life cycle of the virus, enhances epidermal growth factor receptor accumulation and improves MAP kinase activation, and synergistically suppresses p53 expression to induce a carcinogenic effect.^60^

E6 and E7 proteins are oncogenic, which by affecting p53 and pRb, respectively, gradually help to progress toward malignancy by inducing genomic instabilities.^61^ In fact, continued expression of oncoprotein E6 by interfering with the p53 signaling pathway leads to the breakdown of p53 protein, and this is the initiator of malignant changes.^39^, ^62^ The p53 protein is a type of main transducer that concentrates stress signals and transforms them into a set of responses including stopping cell growth, apoptosis, or DNA repair. The activation of p53 after DNA damage or oncogenic signals is a kind of cell protection mechanism.^63^ One of the important functions of this protein is to regulate cell cycle checkpoints and is also responsible for maintaining the integrity of the genome. This protein stops the cell cycle by increasing the expression of p21, p27, p16, and p15 proteins.^64^

Many studies have shown that changes in p53 expression are essential for carcinogenesis and deregulation of p53 is considered a valuable biomarker in the malignant changes of premalignant oral lesions.^65^ E6 and E7 proteins, especially in HPV‐16/‐18, are able to affect the integrity of DNA and chromosome fragments in host cells and also increase the activation of telomerase, which can lead to malignant transformation through infected host cells by promoting cyclin activity of E and A, blocking the functions of interferon, reducing the biological activity of tumor suppressor genes including P53 and retinoblastoma.^7^

Retinoblastoma protein (pRb) plays the role of the cell proliferation controller by stopping the cell cycle in the G1 phase and maintaining chromosomal stability, which is done by stopping E2F transcription factors.^66^ The binding of E7 oncoprotein to retinoblastoma protein pRb disrupts the regulatory pathway of E2F, which is from the group of transcription‐activating factors.^59^ Malignant changes by HPV seem to depend on the type of virus and the physical state of its DNA in terms of integration with Host DNA is dependent. The occurrence of malignant changes requires synergistic actions between HPV and chemical or physical carcinogens as well as genetic instabilities.^21^

However, as some studies show, the results regarding the role of HPV in the pathogenesis of OLP and its malignant changes have been contradictory.^67^ Among the studies included in this review, two studies evaluated the association of HPV with malignant changes in OLP. The first study compared the presence of HPV in dysplastic and non‐dysplastic OLP. Another study evaluated and compared the presence of HPV in nonmalignant OLP and OLP with malignant changes. The authors reported that HPV prevalence was higher in dysplastic than in non‐dysplastic samples. They also stated that the prevalence of HPV in non‐dysplastic OLP is higher than in dysplastic OLP lesions.^20^ The specific role of HPV in the development of OSCC and potentially malignant oral disorders (OPMD) is still debated. Despite the recognition of several factors related to malignant changes, there are still no definite clinical, histological, or molecular factors to predict the development of malignancy in OLP.

A wide variety of assay methods have been used to detect HPV in OLP^68^ (Table 2). Diagnostic methods can also be affected by sample quality. Some recent studies have also found HPV in saliva and exfoliated cells of the mouth, where the sensitivity and specificity of this method is very low^69^ and biopsy is considered a more reliable method to confirm HPV status than saliva analysis. The inherent advantage of the PCR method is its ability to determine a very small amount of HPV DNA, and it is used as a nonquantitative method for HPV DNA. While PCR is often used as a diagnostic method in epidemiological studies of HPV, the economic and technical aspects necessary to apply this method generally prevent its use in large population screening programs. At the same time, laboratory processes and the need for careful control are necessary to reduce false positive results related to contamination in this method.^68^ And the fact that information about the abundance of a specific DNA species is not provided in this method.^70^

IHC is widely used to detect abnormal cells and identify their origin based on the binding of specific antibodies to antigens in the target tissue. The use of IHC in the diagnosis of cervical HPV infections through the detection of p16 expression is considered to be a highly sensitive method.^71^ ISH is also a method to identify specific nucleotide sequences through the binding of a specific nucleotide probe to the target nucleotide sequence in DNA or RNA present in cells. To identify HPV, compared to immunohistochemical staining for p16, the ISH method has been reported with a very high specificity (100%) and a sensitivity of about 80%.^70^ The performance of DNA PCR and DNA ISH is comparable to each other, but a direct comparison of the two shows that DNA ISH may be more useful as a diagnostic tool due to its ability to reliably detect HPV infection from a passenger virus.^71^ Among the articles examined in this review study, only three studies used the ISH method to identify HPV DNA, and a lower prevalence of HPV was reported in these articles than others. It seems that more studies are needed to accurately investigate the prevalence of HPV in OLP using the ISH method.

Despite extensive studies regarding the role of HPV in oral cavity lesions, there are still no specific clinical guidelines for testing for HPV infection in oral lesions. In terms of the type of diagnostic method, according to Table 2, among the articles reviewed in this review study, 14 studies were conducted on fresh biopsy of the oral mucosa and 10 studies on FFPE blocks and 1 study has been conducted with the brushing method of oral mucosa cells. Among these studies, the prevalence of HPV in studies that used FFPE paraffin blocks has been reported from 1% to 30%, which reported a lower prevalence compared to fresh biopsies from the oral mucosa (HPV prevalence from 7.1% to 86.6%). FFPE paraffin blocks are considered as efficient and available sources for identifying HPV genotypes and histological classifications, but DNA isolation in FFPE samples is a challenging process because the length of the isolated DNA strand in this method is very important to achieve a reliable result in PCR. On the other hand, several factors are involved in DNA damage in fixed paraffin blocks. The fixation time, especially more than 12–24 h, the use of very acidic stabilizers, long storage periods, and storing the samples in hot environments can all lead to the destruction of DNA residues in the samples, and these technical differences decrease the sensitivity of this method in identifying HPV.^72^ It seems that in conducting more detailed studies, the use of a fresh biopsy method from the oral mucosa (fresh biopsy) is preferable to paraffin blocks, according to the mentioned materials.

Regardless of the numerous articles that have investigated the relationship between HPV and OPMD, there are few articles related to the mechanism of interaction between HPV and OPMD. To date, two hypotheses have been proposed regarding the cause of the susceptibility of potentially malignant oral lesions to HPV infection. The first theory is that the presence of an ulcer in the mucosa can make the oral cavity more susceptible to HPV, and this is why EA‐OLP and inhomogeneous leukoplakia are more associated with HPV.^73^ The second theory refers to the role of steroids in increasing the proliferation of this virus. Since the first line of treatment in symptomatic OLP is the use of immunosuppressive drugs, especially corticosteroids, which relieve symptoms, the use of immunosuppressive drugs in the management of OLP may increase viral replication in the oral cavity with mucosal atrophy. Chronic use of topical steroids with high strength suppresses the immune system and as a result HPV proliferation is upregulated.^15^ Topical steroids are effective in the proliferation of HPV in the mucosa by reducing immune cells in the oral mucosa, including monocytes and lymphocytes, as well as reducing the secretion of inflammatory cytokines, including IL‐1, IL‐6, and TNF‐α. IL‐1 and TNF‐α are selective suppressors of DNA transcription in HPV.^74^ This ability of topical steroids to stimulate viral expression was discussed in a study on 20 men treated with topical steroids who had genital lichen planus lesions and HPV was detected in them. High‐risk HPV was detected in 16% of the sample and low‐risk HPV was not detected in any of the samples. HPV expression increased up to 21% following treatment with topical steroids^75^ and the authors emphasized the need for close monitoring in patients. In none of the studies reviewed in this review study, the prevalence of HPV in OLP samples before and after treatment with corticosteroid drugs has not been evaluated, and more studies are needed in this field.

The findings of this review study show that the prevalence of oral HPV infection is higher in men than in women. The rate of orogenital HPV transmission from women to men has been reported to be higher than other methods. A reasonable theory is that the risk of oral HPV infection in men is higher among heterosexuals than among homosexuals, and a weaker immune response against HPV occurs in men.^76^ Some researchers stated that men have a higher prevalence of HPV‐16 infection than women in oral rinse samples, and this may indicate a sex‐specific predisposition to HPV‐16 infection.^77^ In addition, the higher seroconversion rate in women may be related to their stronger protection against extragenital HPV infections.^78^ This phenomenon can explain the fact that in several developed countries around the world, the incidence of HPV‐positive oral and pharyngeal cancers among men has increased significantly during the last three to four decades, while this rate has increased moderately among women^79^.

The prevalence of HPV in OLP varies based on different parameters such as geographical differences in the population, HPV genotype, clinical type of OLP, and HPV identification method. There are considerable differences in the geographical distribution of HPV in OLP in different regions of the world. Among the studies examined in this review, only two studies were conducted with the aim of investigating the prevalence of HPV in OLP patients, considering the geographical distribution. In the first study, the authors stated that the prevalence of HPV in the Thai population with OLP is low.^16^ In another study conducted in Iran, HPV DNA was detected in 27.5% of cases of OLP patients^27^.

## 5 CONCLUSION

The conclusion of this review study is as follows:

1. The most common HPV genotypes reported in OLP are genotypes 16 and 18.
2. In terms of virus detection methods, PCR and IHC methods have been widely used in the articles, but few studies are using the ISH method.
3. In terms of the type of examined samples, it is recommended that future studies be performed using fresh biopsy from the oral mucosa.
4. In none of the studies examined in this review, the prevalence of HPV virus in OLP, before and after treatment with topical steroids, has not been investigated.
5. The prevalence of HPV in OLP has not been widely discussed in terms of the clinical type of OLP lesions, and further studies are recommended to investigate the relationship between this virus and the clinical types of OLP.

Despite the studies conducted on the relationship between OLP and HPV infection, there is still no conclusive evidence that HPV can play a role in the etiopathogenesis of OLP, either in clinical manifestations or in the malignant transformation of lesions. The association between HPV infection and OLP should be interpreted with caution because it is not a constant phenomenon and could be just an incidental finding. More prospective cohort studies are needed to demonstrate the potential role of HPV infection in the incidence of OLP.

## AUTHOR CONTRIBUTIONS

**Farzaneh Agha‐Hosseini**: Conceptualization; investigation; project administration; supervision. **Kimia Hafezi Motlagh**: Conceptualization; data curation; formal analysis; investigation; methodology; writing—original draft; writing—review and editing.

## CONFLICT OF INTEREST STATEMENT

The authors declare no conflict of interest.

## Supporting information

Supporting information.

## Data Availability

The data that support the findings of this study are available on request from the corresponding author.
